# Mapping terrestrial oil spill impact using machine learning random forest and Landsat 8 OLI imagery: a case site within the Niger Delta region of Nigeria

**DOI:** 10.1007/s11356-018-3824-y

**Published:** 2018-12-07

**Authors:** Mohammed S. Ozigis, Jorg D. Kaduk, Claire H. Jarvis

**Affiliations:** 1 Department of Geography, University of Leicester, Leicester, United Kingdom; 2 Department of Strategic Space Applications, National Space Research and Development Agency (NASRDA), Abuja, Nigeria

**Keywords:** Oil spill, Vegetation health indices, Spectral bands, Random forest, Variable importance, Landcover

## Abstract

Terrestrial oil pollution is one of the major causes of ecological damage within the Niger Delta region of Nigeria and has caused a considerable loss of mangroves and arable croplands since the discovery of crude oil in 1956. The exact extent of landcover loss due to oil pollution remains uncertain due to the variability in factors such as volume and size of the oil spills, the age of oil, and its effects on the different vegetation types. Here, the feasibility of identifying oil-impacted land in the Niger Delta region of Nigeria with a machine learning random forest classifier using Landsat 8 (OLI spectral bands) and Vegetation Health Indices is explored. Oil spill incident data for the years 2015 and 2016 were obtained from published records of the National Oil Spill Detection and Response Agency and Shell Petroleum Development Corporation. Various health indices and spectral wavelengths from visible, near-infrared, and shortwave infrared bands were fused and classified using the machine learning random forest classifier to distinguish between oil-free and oil spill–impacted landcover. This provided the basis for the identification of the best variables for discriminating oil polluted from unpolluted land. Results showed that better results for discriminating oil-free and oil polluted landcovers were obtained when individual landcover types were classified separately as opposed to when the full study area image including all landcover types was classified at once. Similarly, the results also showed that biomass density plays a significant role in the characterization and classification of oil contaminated and oil-free pixels as tree cover areas showed higher classification accuracy compared to cropland and grassland.

## Introduction

An oil spill is the discharge of petroleum hydrocarbon products into marine or terrestrial ecosystem. Terrestrial spills result from underground and surface pipeline leakages, sabotage, and operational failure, as well as transport of oil slicks from sea to land (Taheri 2012). Oil can damage vegetation through several mechanisms, such as the ingestion and absorption of toxic compounds through the biota’s respiratory structures (Joel and Amajuoyi 2009; Mendelssohn et al. 2012), coating and smothering which affects temperature adaptation, and gas regulation as well as other life-supporting processes (Mendelssohn et al. 2012). On shore, oil spill contamination has the potential of increasing erosion and loss of salt marsh due to oil-induced plant mortality (Khanna et al. 2013) and the longer oil resides on land, the greater the impact and slower the recovery (Gundlach and Hayes 1978; Jackson et al. 1989; Khanna et al. 2013). This results from direct impacts of hydrocarbon crude oil on plant metabolism as well as indirect impacts through disruption of plant-water relationships and reduced gas exchange between atmosphere and soil (Hester and Mendelssohn 2000; Khanna et al. 2013; Pezeshki et al. 2000).

In Nigeria, the effects of oil exploration are particularly glaring in the Niger Delta. Reduced food productivity, damages to the subsistence economy, habitat distortion, epidemic outbreaks, and general social instability are among the numerous negative impacts that crude oil exploitation has had in the Niger Delta (Onwurah et al. 2007). The Nigerian Conservation Foundation in a study in 2006 put the figure for oil spilt, onshore and offshore, at 9 to 13 million barrels of oil over the past 50 years. This has massively threatened the well-being of the people (Nriagu 2011). Onwurah et al. (2007) noted that a good percentage of oil spills that occurred on the dry land between 1978 and 1979 in Nigeria affected farmlands in which crops such as rice, maize, yams, cassava, and plantain were lost. Similarly, findings from the studies conducted by the United Nation Environmental Programme (UNEP) in 2011 in the Niger Delta suggest that residents are exposed to elevated levels of petroleum hydrocarbon in contaminated drinking water and outdoor air which posed a serious threat to their health (UNEP 2011).

Detecting oil spill through remote sensing is frequently the basis for establishing the impact of oil pollution near shore, marshes, and mudflat ecosystems. Common techniques used for oil spill detection include image spectroscopy (Khanna et al. 2013; Kokaly et al. 2013) and field spectroscopy (Mishra et al. 2012), broadband Vegetation Health Indices (Adamu et al. 2015; Arellano et al. 2015; Noomen et al. 2015), narrowband vegetation indices (Arellano et al. 2015; Noomen et al. 2015), and recently airborne SAR polarimetry (Ramsey et al. 2015; Ramsey III et al. 2011; Ramsey et al. 2014). Results from satellite image processing with emphasis on vegetation health are particularly useful in assessing the impact of oil on terrestrial mangrove and swamp ecosystems as well as fragile near-shore marsh vegetation (Adamu et al. 2016; Khanna et al. 2013; Kokaly et al. 2013; Mendelssohn et al. 2012; Mishra et al. 2012; Noomen et al. 2015; Onwurah et al. 2007; Ramsey et al. 2015; Ramsey III et al. 2011; Shi et al. 2007; Sun et al. 2016; Zabbey and Uyi 2014). This is because of the toxicity of crude oil and its potential to alter the biophysical and biochemical processes in plants and ecosystem community. However, most studies in oil spill impact assessment have focused on detecting the phenomenon without necessarily establishing the extent of the impact of these obnoxious compounds on the adjoining landcover. Attempts have also been made to map landcover changes as a result of the long-term impact of hydrocarbon on plant communities (Ayanlade and Howard 2016; Kuenzer et al. 2014; Ochege et al. 2017). A significant number of studies have primarily focused on assessing general changes on mangrove fields over time without specific efforts to distinguish between the healthy components (oil-free) and oil-impacted landcover component, and how the observed trends affect the broader landcover change.

This study focuses explicitly on distinguishing and mapping oil-free and oil-impacted landcovers separately. This can provide a basis for assessing future terrestrial based oil spill impacts and how the inter landcover variability of oil polluted and oil-free landcover types contribute to a general landcover change pattern. Furthermore, the effective discrimination of oil polluted and oil-free landcovers can provide information on the location of oil pipeline leakages and the extent of land area affected by oil in regions with limited accessibility. This mapping can also provide useful landcover discriminatory maps for timely intervention in oil spill prone areas, as well as a basis for formulating mitigation and remediation strategies before irreversible damage is done to the ecosystem. In the long term, however, this approach can also be used to formulate robust and transferable image processing models which can be used to track future terrestrial oil spills leveraging on the pool of spectral library generated.

Some studies have tried to reduce the confusion between classes by implementing spectral space delineation to obtain pure image training samples specific to each class to generate accurate maps (Aplin and Atkinson 2001; Arif et al. 2015; Arroyo et al. 2010; MacLachlan et al. 2017; Tsutsumida et al. 2016).

Generally, two fundamental types of image processing methodologies exist, parametric and non-parametric algorithms (Li et al. 2013). While the first is dependent on the characteristic nature of input variables with respect to statistical distribution, probability, and clustering of pixel values, the non-parametric methods do not require variables to follow a particular statistical distribution and they also have the ability of discretely handling problems of noise, model fitting, and relatively lower computational demands than other classification approaches. Several on shore oil spill studies have used decision tree algorithms for the assessment of oil contamination on mangrove and marshland. Giri et al. (2011) used a decision tree classifier based on a univariate decision tree (C45.5) algorithm to classify Landsat and Airborne photography of the Louisiana mangroves. Emphasis was on depicting the spatiotemporal characteristics of ecosystem shifts, in terms of expansion, retraction, and disappearance. Khanna et al. (2013) also used a binary decision tree based on vegetation index, angle index, and depth of oil absorption to produce a classification map for six classes, oiled soil, oiled dry vegetation, oil-free soil, oil-free dry vegetation, green vegetation, and water to assess oil impact on marshland vegetation of the Louisiana coast. However, little attempts have been made to assess the functionality of random forest classification algorithms for discriminating oil-impacted landcover from oil-free landcover at a broader scale. The robust application of random forest in the extraction of precise details from remotely sensed data has been demonstrated in several studies (Du et al. 2015; Jhonnerie et al. 2015; Juel et al. 2015; Liu et al. 2014).

This study aims to

- Explore the potential of the non-parametric random forest machine learning classifier to discriminate pixels of oil polluted landcover from oil-free landcover types within the Niger Delta region of Nigeria using Landsat 8 visible, near-infrared, and shortwave infrared bands and derived Vegetation Health Indices
- Identify the variables that provide most information for this discrimination using this non-parametric method, as several studies (Adamu et al. 2015, 2016, 2018; Khanna et al. 2013; Zhu et al. 2013) have tested the sensitivity of some of these variables to detect oil spill using parametric methods
- Highlight the possible reduction of confusion between classes by implementing subset classification for the separate landcover types of cropland, grassland, and tree cover areas is demonstrated

## Materials and methods

### The study area

**Fig. 1:**
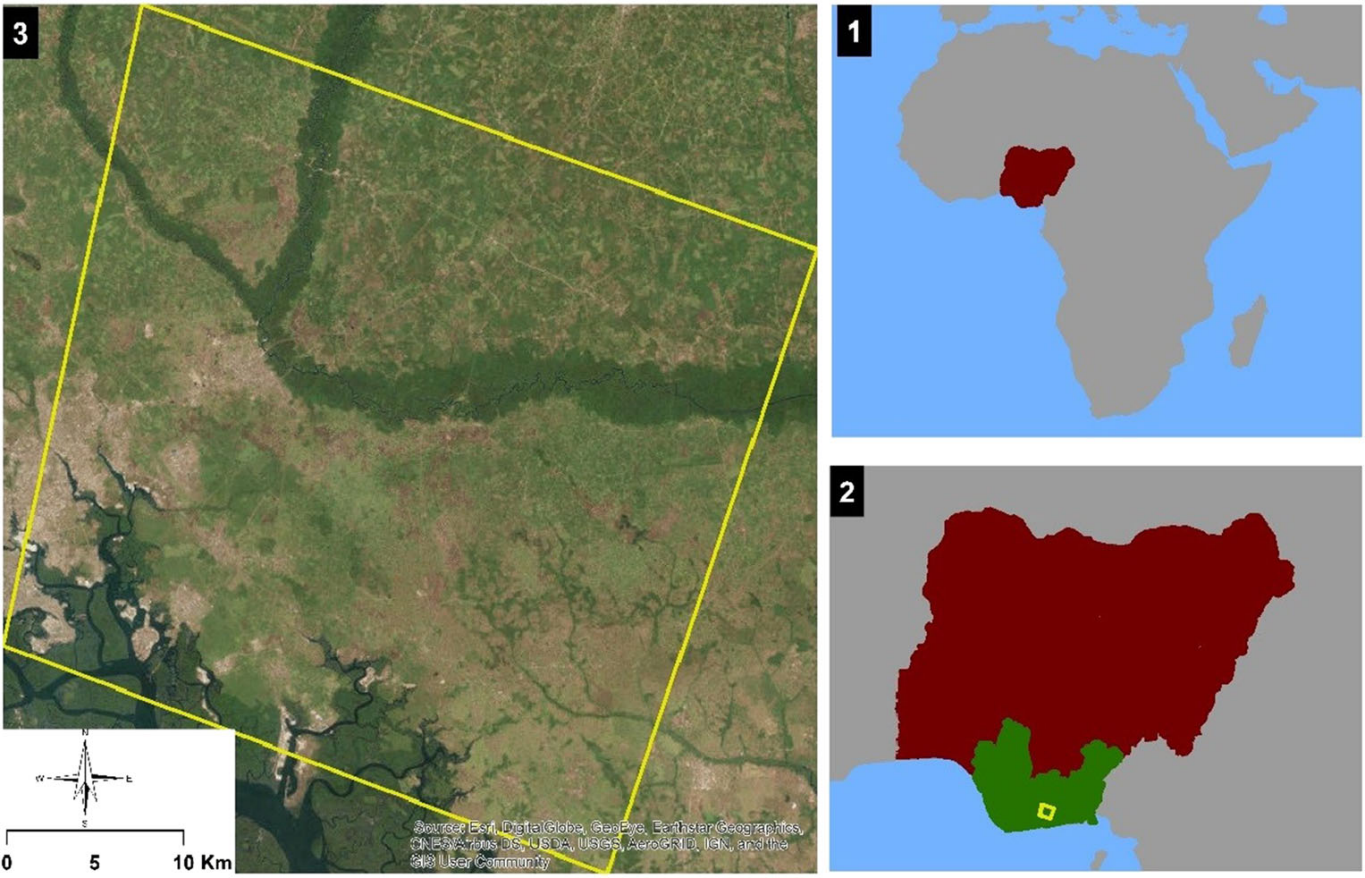
Location of the study area within Nigeria in Africa. The brown-colored area represents Nigeria within the African continent, while the green-colored area is the oil-producing Niger Delta region. The image shows the precise extent of the study area (Source: ESRI, ArcGIS Base Map Image, provided by Digital Globe, GeoEye, and Airbus)

The study area defined by four corner coordinates of longitude 6.957° E latitude 5.025° N, longitude 7.247° E latitude 5.025° N, longitude 6.96° E latitude 4.795° N, and longitude 7.254° E latitude 4.804° N covers 1320 km^2^ within the Niger Delta region of Nigeria (Fig. 1). It cuts across Abia and Rivers States. To the far west corner is the Ukwa West Local Government Area of Abia State and to the easterly corner are Ikwerre, Obio/Akpor, Eberi/Omumma, Oyigbo, Eleme, and Port Harcourt Local Government Area of Rivers state.

### Data

Three datasets were used in this research: oil spill incident data, satellite image (Landsat 8, Operational Land Imager), and the landcover data.

#### Oil spill incident data

The oil spill dataset was obtained from two published sources, the Shell Petroleum Development Corporation (SPDC) https://www.shell.com.ng/sustainability/environment/oil-spills.html and the National Oil Spill Detection and Response Agency (NOSDRA) https://oilspillmonitor.ng/. The NOSDRA is a government agency tasked with capturing all oil spill incidents both in marine and terrestrial realms across the country.

#### Landcover data

The landcover map for the African continent produced by the European Space Agency Climate Change Initiative 2016 was used in this study (http://2016africalandcover20m.esrin.esa.int/). The product contains 10 classes for different landcover categories including built-up areas, waterbody, and various vegetation types produced from 20-m-high spatial resolution Sentinel-2A image over Africa. The tile information covering the study area was downloaded, subsetted, and used for the establishment of appropriate landcover types for the study area. The major landcover categories used in this study were cropland, grassland, and tree cover areas (TCA). Features such as built-up areas, waterbody, and baresurface were excluded from this study as most oil pipelines and the corresponding spill incidents occur on terrestrial vegetation classes. Thus, their exclusion reduced artifacts and misclassification.

#### Landsat 8: OLI image

The Landsat 8 (OLI data) for the year 2016 was downloaded from the USGS website (earthexplorer.usgs.gov). The image acquired was a Landsat surface reflectance higher-level data product processed using the Landsat surface reflectance code (LaSRC). The LaSRC makes use of the coastal aerosol band to perform aerosol inversion tests using auxiliary climate data from MODIS and a unique radiative transfer model (Roy et al. 2014). Additionally, LaSRC hardcodes the view zenith angle to “0” and solar zenith which are used for calculations as part of the atmospheric correction process. The image acquired and used for this study, acquired on the 6th of December, is a post spill dry season image with little to no cloud cover, aerosol, and haze effect. Images between the months of March and November had significant cloud cover due to the wet season.

### Methods

#### Sampling regime

- Spill incident harmonization

The oil spill data harmonization sought to integrate and expand the oil spill database for this research. The harmonization operation was carried out by overlaying both datasets (NOSDRA and SHELL) in a GIS environment. Points with repeated information as a result of duplicate capture and multiple spill incidents over the years were identified and marked. Duplicates (in most cases the SPDC data) were deleted since the dataset provided by NOSDRA is all encompassing as the government’s regulatory agency with the responsibility of documenting all spill incidents. The spill information relating to volume, size, and date of spill was checked, as this provided the basis for tracking the spill intensity on the different landcover types. The minimum area covered by the spill data used for this exercise is 1000 sqm, which is greater than a single Landsat image pixel of 900 sqm. This is to ensure that pixels used for training, testing, and validation of the final model as well as the image classification have dominant spectral reflectance of a typical oil polluted site.

- Assignment of spill incidents to landcover

The assignment of oil spill incidents to the corresponding landcover categories is an important step in this study, as the *RF* algorithm would rely on the spectral signatures provided by these training sites to build a robust model. For each landcover class (cropland, grassland, and TCA), spill incidents located within the landcover classes were identified. This provided the various training and validation sites for the identification of oil-impacted (polluted landcover) classes.

- Selecting non-polluted sites for the different landcover

Non-polluted sample sites are necessary in this study for two main reasons: first, for the identification of oil-free (non-polluted) landcover types within the study area and secondly for an effective discrimination between pixels of oil-free and oil spill–impacted landcovers. Proximity analysis as suggested by (Obida et al. 2018; Park et al. 2016; Whanda et al. 2016) provided the basis for the selection of the polluted and oil-free vegetation pixels. The minimum rule was set that all non-polluted sites must be located at least 600 m away from all polluted sites based on the maximum area of spill recorded. This resulted in an 800 m buffer ring around all existing spill points, which avoided any overlap with any likely spill-impacted area. The procedure ensured that sample sites selected for the respective oil-free landcover are reasonably well-spaced from the oil polluted sites. Thereafter, the training sites for the non-polluted landcover categories were selected at random outside the buffer ring established. Furthermore, specifically only healthy vegetation as inferred from high-resolution Google Earth image was chosen.

- Pixel selection using buffer analysis

Following the reconciliation and extraction of the oil spill points and the non-polluted sites respectively according to their respective landcover classes (cropland, grassland, and TCA), the points were then sub-divided into two categories for training and validation purpose. Sixty percent of the points for individual landcover category were randomly selected for training, while the other 40% were set aside for validation in post classification accuracy assessment. Table 1 shows the distribution of the polluted spill sites and oil-free sites according to their respective landcover classification schemes. To this end, 30 m buffer ring polygons were established around all the training sites to ensure that only adjacent pixels within the high consequence area close to the point of impact are selected specially for the polluted sites (Alexakis et al. 2016; Whanda et al. 2016).

**Table 1:** Total number of sites used for calibrating and validating the random forest classification

Class label	Number of spill sites
Non-polluted cropland	41
Non-polluted grassland	27
Non-polluted tree cover areas	25
Polluted cropland	44
Polluted grassland	26
Polluted tree cover areas	26

#### Image preprocessing

As the Landsat surface reflectance higher-level data product was obtained, there was no need to carry out any atmospheric correction operations.

- Geometric correction

In order to ensure that the Landsat 8 (OLI satellite image) co-registers properly with the other datasets (such as the oil spill sites and boundary dataset), the satellite image was re-projected to the Universal Transverse Mercator projection and the World Geodetic Survey 1984 Datum of Zone 32 North (UTM WGS84 Zone 32N).

- Landcover image masking

Following the geometric correction of the study area image, the three dominant existing landcover classes extracted from the ESA CCI data (“Landcover data”) were used to subset the image for the different landcover types. This provided the basis of implementing a general study area wide classification operation (at macro level) and individual landcover subset classification (at micro level). The landcover image extent generated was for cropland, grassland, and TCA (i.e., dense canopy vegetation), in which the harmonized oil spill and oil-free landcover training sites were used to implement a macro and micro level classification. This produced six different landcover schemes, that is, polluted (oil-impacted) cropland, polluted grassland, polluted TCA, non-polluted (oil-free) cropland, non-polluted grassland, and non-polluted TCA.

#### Retrieval of important Vegetation Health Indices

Eight Vegetation Health Indices were generated using the formulae presented in Table 2. The indices were generated from the pre-processed Landsat 8 (OLI image) of the study area using the red, green, blue, near-infrared, shortwave infrared 1, and shortwave infrared 2 bands.

**Table 2:** Vegetation Health Indices generated using the red, green, blue, NIR, and SWIR bands

Vegetation indices	Formula	Author
Difference Vegetation Index	*R*_NIR_ − *R*_RED_	Tucker 1980
Modified Soil-Adjusted Vegetation Index	$1/2\left[2{R}_{\mathrm{NIR}}+1-\sqrt{\left(2{R}_{\mathrm{NIR}}+1\right)-8\left({R}_{\mathrm{NIR}}-{R}_{\mathrm{RED}}\right)}\right]$	Qi et al. 1994
Moisture Stress Index	*R*_MidIR_/*R*_NIR_	Doraiswamy and Thompson 1982
Normalized Difference Vegetation Index	(*R*_NIR_ − *R*_RED_)/(*R*_NIR_ + *R*_RED_ )	Rouse Jr et al. 1974
Normalized Differential Water Index	(*R*_NIR_ − *R*_SWIR_)/(*R*_NIR_ + *R*_SWIR_)	Hardisky et al. 1983
Renormalized Difference Vegetation Index	${R}_{\mathrm{NIR}}-{R}_{\mathrm{RED}}/\sqrt{R_{\mathrm{NIR}}+{R}_{\mathrm{RED}}}$	Roujean and Breon 1995
Ratio Vegetation Index	*R*_RED_/*R*_NIR_	Jordan 1969
Soil and Atmospherically Resistant Vegetation Index	(1 + 0.5) (*R*_NIR_ − *R*_RB_)/(*R*_NIR_ + *R*_RB_ + 0.5)	Qi et al. 1994
Soil-Adjusted Vegetation Index	(1 + *L*)(*R*_NIR_ − *R*_RED_)/(*R*_NIR_ + *R*_RED_ + *L*)	Huete 1988
Transformed Difference Vegetation Index	$\sqrt{R_{\mathrm{NIR}}-{R}_{\mathrm{NIR}}}/\Big(\left({R}_{\mathrm{NIR}}+{R}_{\mathrm{RED}}\right)$+ 0.5)	Bannari et al. 2002

#### Random forest classifier

The random forest (*RF*) algorithm was proposed by Breiman (2001). It is an ensemble method for supervised classification and regression, based on classification and regression trees (CART). It relies on the assumption that different independent samples can influence positive predictions in different areas, thus combining these true positives can significantly improve overall prediction accuracy (Polikar 2006). The method also seeks to optimize training samples by randomly selecting samples to split each node in the decision trees to maximize prediction accuracy. This offers the opportunity of including many variables in a single classification operation, which in turn should contribute positively to the prediction of the final class. A list of variable importance and their contribution toward class assignment during the classification process is generated through the mean decrease in Gini (MDG) coefficient. The *RF* classification was used to distinguish and effectively characterize landcover impacted by oil pollution from oil-free vegetation. The analysis was carried out using the ImageRF component of the EnMap Box (Waske et al. 2012). To achieve this, various Vegetation Health Indices (generated in “Sampling regime”) together with seven Landsat (8 OLI bands) (across visible, NIR, and SWIR) were fused for the classification process. The tree size (*ntree*) used for classification was determined through repetitive runs before an optimal value of 500 (*ntree*) was arrived at and used for parametrization in all classification scenarios implemented. Table 3 outlines the list of variables used for the *RF* classification.

**Table 3:** List of variables used for the *RF* classification

S/no	Spectral variables
1	Band 1—ultra-blue band
2	Band 2—blue
3	Band 3—green
4	Band 4—red
5	Band 5—near-infrared (NIR)
6	Band 6—shortwave infrared (SWIR) 1
7	Band 7—shortwave infrared (SWIR)2
8	Difference Vegetation Index (DVI)
9	Modified Soil-Adjusted Vegetation Index (MSAVI)
10	Moisture Stress Index (MSI)
11	Normalized Differential Vegetation Index (NDVI)
12	Normalized Differential Water Difference (NDWI)
13	Renormalized Difference Vegetation Index (RDVI)
14	Ratio Vegetation Index (RVI)
15	Soil and Atmospherically Resistant Vegetation Index (SARVI)
16	Soil-Adjusted Vegetation Index (SAVI)
17	Transformed Normalized Difference Vegetation Index (TNDVI)

#### Accuracy assessment

Two performance indicators were employed to assess the *RF* calibration model and the resulting classified image obtained. First is the F1 accuracy, which is the harmonic mean of precision and sensitivity (recall) accuracy statistics. This is used in the ImageRF to assess the out of bag error of the *RF* calibration. The precision is the ratio of correctly predicted positive pixels to the total positive observations (incorporating true positives and false positives), while the recall is the ratio of correctly predicted positive observations to the sum of true positives and false negative observations. This however can be further interpreted as the measure of truly assigned pixels to a particular class (recall) and the measure of truly assigned pixels in the image space. The F1 score is a robust accuracy measure for model performance. This is because it seeks to balance the influence of recall and precision through the use of harmonic mean of both measures.

This is denoted by the formulae below:

1 $$ F1\  Accuracy=2\times \frac{Precision\times Recall}{Recall+ Precision} $$

2 $$ Precision=\frac{TP}{TP+ FP} $$

3 $$ Recall=\frac{TP}{TP+ FN} $$

where

TP = true positives

FP = false positives

FN = false negatives

The error matrix as described by (Congalton 1991) was also used to assess the classified image output from the *RF* classification using the 40% validation points (“Sampling regime”). This enabled an effective comparison of the classified image outputs to the original reference sites. Specific attention was given to the users, producers and the overall accuracies.

## Results

### RF model calibration

**Fig. 2:**
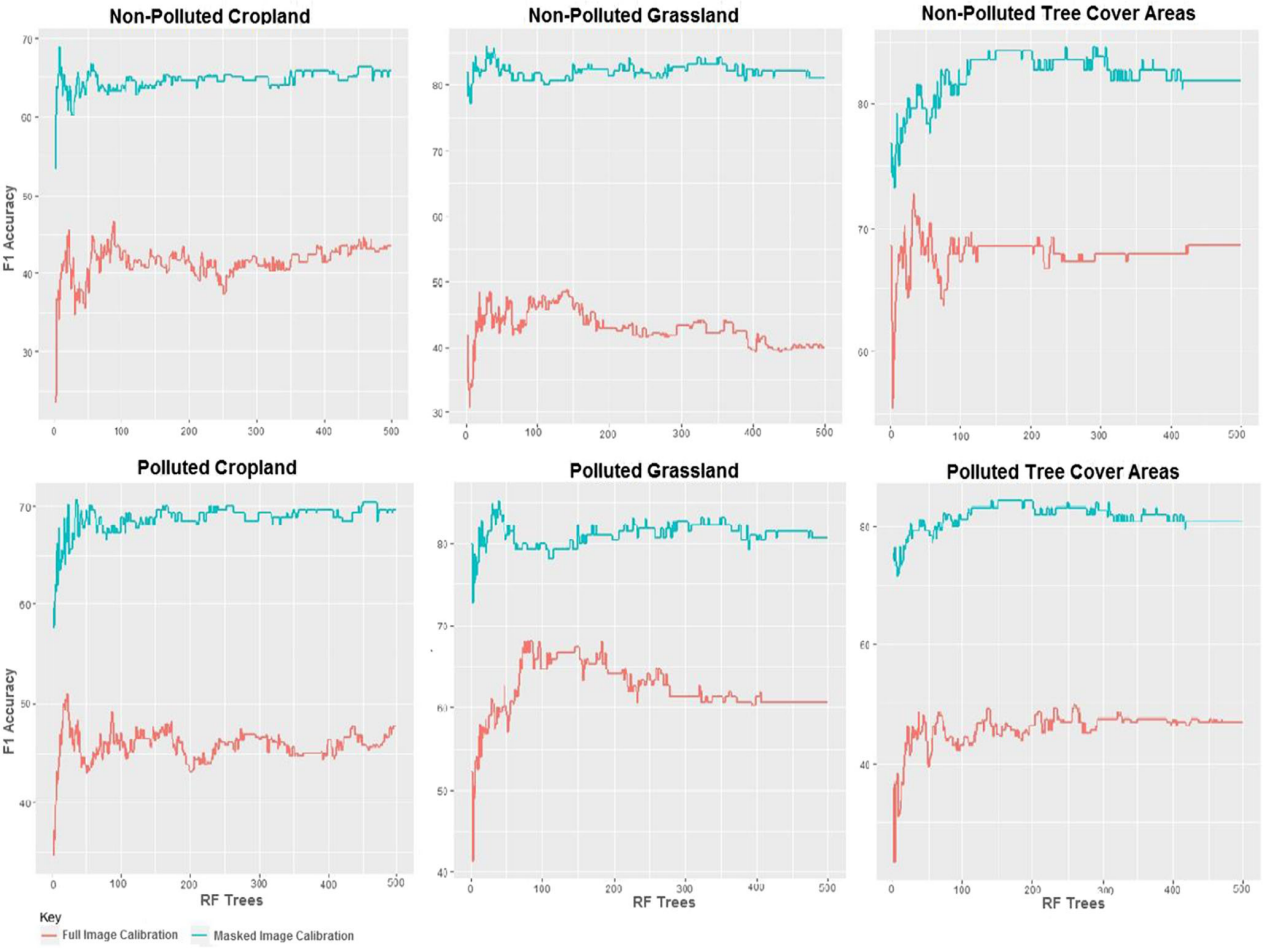
*RF* parameterization result for the full study area image and individual landcover subset images using training samples of oil-free and oil-impacted landcover. The green line represents parameterization F1 accuracy for the individual landcover subset images, while the red line represents F1 accuracy for the full study area image

Figure 2 shows the result of the *RF* out of bag error. In general, the result indicates that the landcover subset images had lower out of bag errors and consequently higher calibration accuracy, compared to the result obtained from the full image calibration. This shows that of the six schemes calibrated, the non-polluted (NP) and polluted (P) TCA and grassland respectively had better calibration result ranging from 45 to 70% F1 accuracy. While on the contrary, both the P and NP croplands had lower calibration accuracies when the full study area image was calibrated. The model calibration result also showed that of the six different schemes investigated, the NP grassland and NP TCA had the best prediction to error ratio of 86% and 84% as indicated in the F1 accuracy when the respective landcover subsets were used. In contrast, the P and NP croplands had the least calibration accuracy. In terms of the implication for interclass separability and model fit, it is observed that calibration accuracy increased gradually from zero and mostly attained saturation when the tree size (*ntree*) in the *RF* reached 50 using the variables, although for some cases the F1 accuracy increased up to 100 trees before maximum saturation was reached. This however implied that a lower *ntree* value could yield sufficient calibration result.

### Landcover subset vs full image classification

**Fig. 3:**
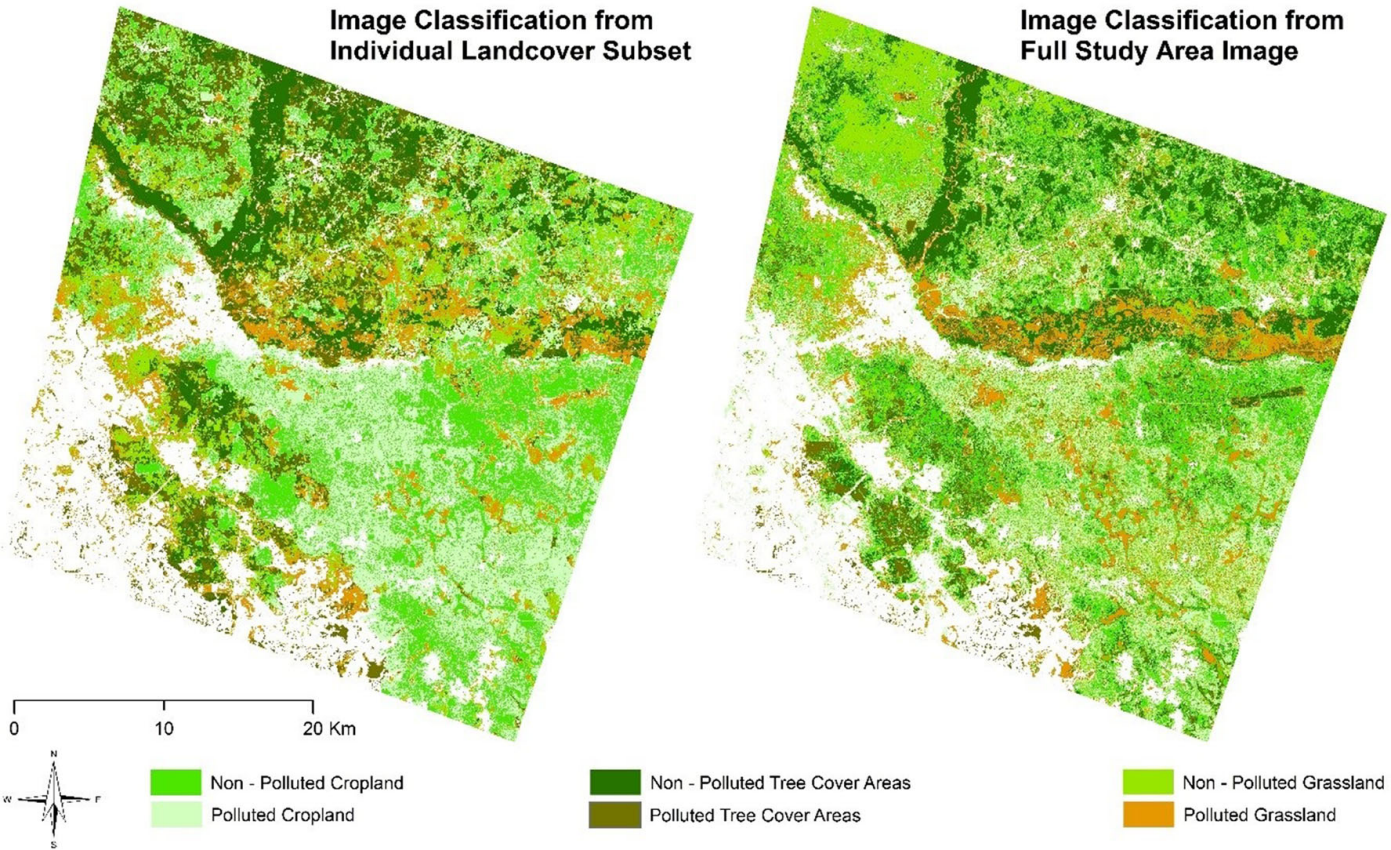
*RF* Image classification result for the full study area image and individual landcover subsets. It is observed that the former produced a more generalized representation of landcover extents compared to the crisp output from the full study area image

**Fig. 4:**
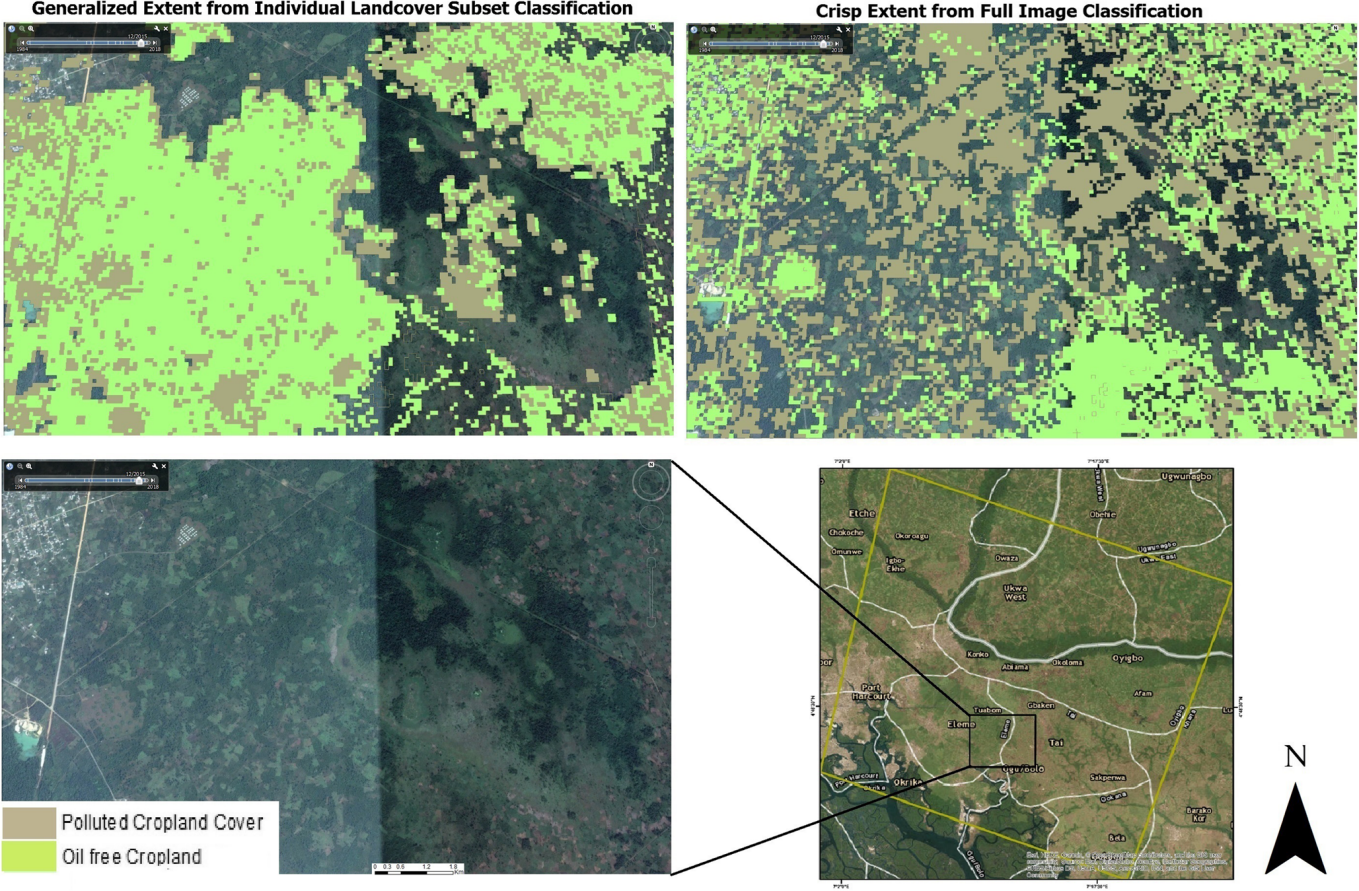
Subset of the study area showing the *RF*-classified image with the landcover subset of cropland into polluted and oil-free croplands. Inset is a high-resolution image from Google Earth for the same area. This showed that spill-impacted and oil-free croplands were better captured by the image subset classification (left), compared to the more crisp extent from the full study area image classification (right)

Figures 3 and 4 show the images classified from the two scenarios. The image classification at the landcover subset level had better representation of landcover extents with a more generalized boundary compared to the full image classification which had a crisper and noisy representation. This however supports various assertions in several studies where subpixel classification has been implemented (Aplin and Atkinson 2001; Arif et al. 2015; MacLachlan et al. 2017). A major reason for the observed disparity could be as a result of the presence of multiple signatures from conflicting landcover features causing high spectral mixing for the *RF* classifier at the macro level. Fröhlich et al. (2013) have also observed that textural characteristics of neighboring adjacent features can inadvertently cause false representation of image features. Similarly, the spectral diversity of the features investigated (polluted and non-polluted landcovers) had smaller separability index as observed from the out of bag error for the full study area image. This can affect the performance of the classifier in adequately producing generalizable extents. The implication of this effect was further assessed using error matrices generated.

### Variable importance

**Fig. 5:**
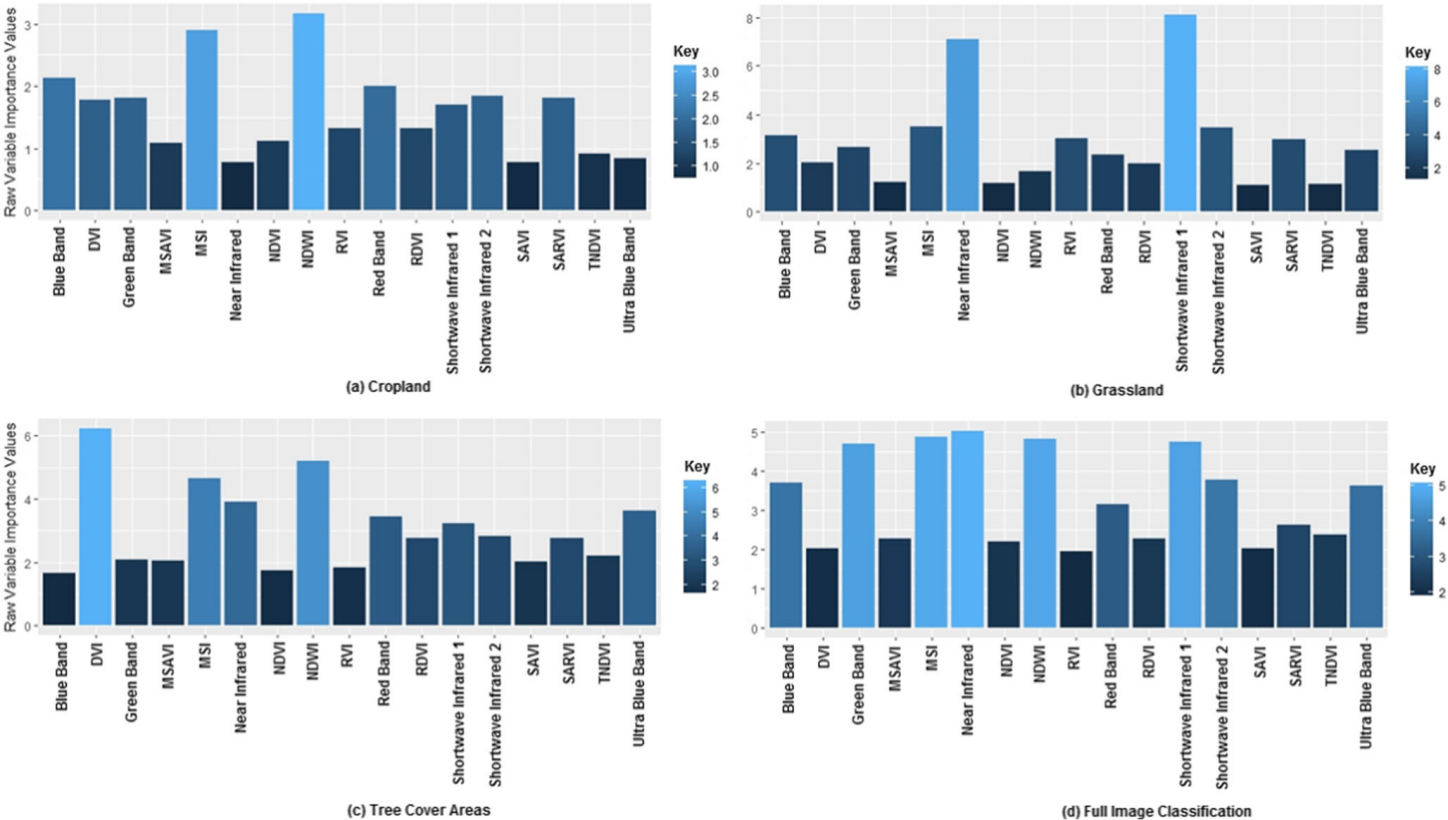
Variable importance plot of the *RF* classification for the full study area image and landcover subset image classification

The near infra band had the highest contribution to the assignment of endmember classes for the six landcover schemes when the full study area image was classified (Fig. 5). Other variables however such as Moisture Stress Index, Normalized Difference Water Index, shortwave infrared 1 (mid infrared region), and the green band also contributed substantially in the classification process. At the subset level, the result showed that the Normalized Difference Water Index and Moisture Stress Index were very influential in providing the best splits between polluted and oil-free cropland landcover schemes. This conforms with results obtained in Kalubarme and Sharma (2015) where NDWI values were observed to be sensitive to stress conditions in wheat-cultivated farm plantations. Similarly, results obtained by Benabdelouahab et al. (2015) also showed that MSI and NDWI are sensitive indicators of stress also in a wheat-cultivated farm field. However, the near-infrared and shortwave infrared bands were also observed to have the highest contribution in splitting oil contaminated and oil-free grassland landcover scheme. While the Difference Vegetation Index (DVI) and Normalized Differential Water Index clearly had strong contribution in splitting oil polluted from oil-free TCA.

**Fig. 6:**
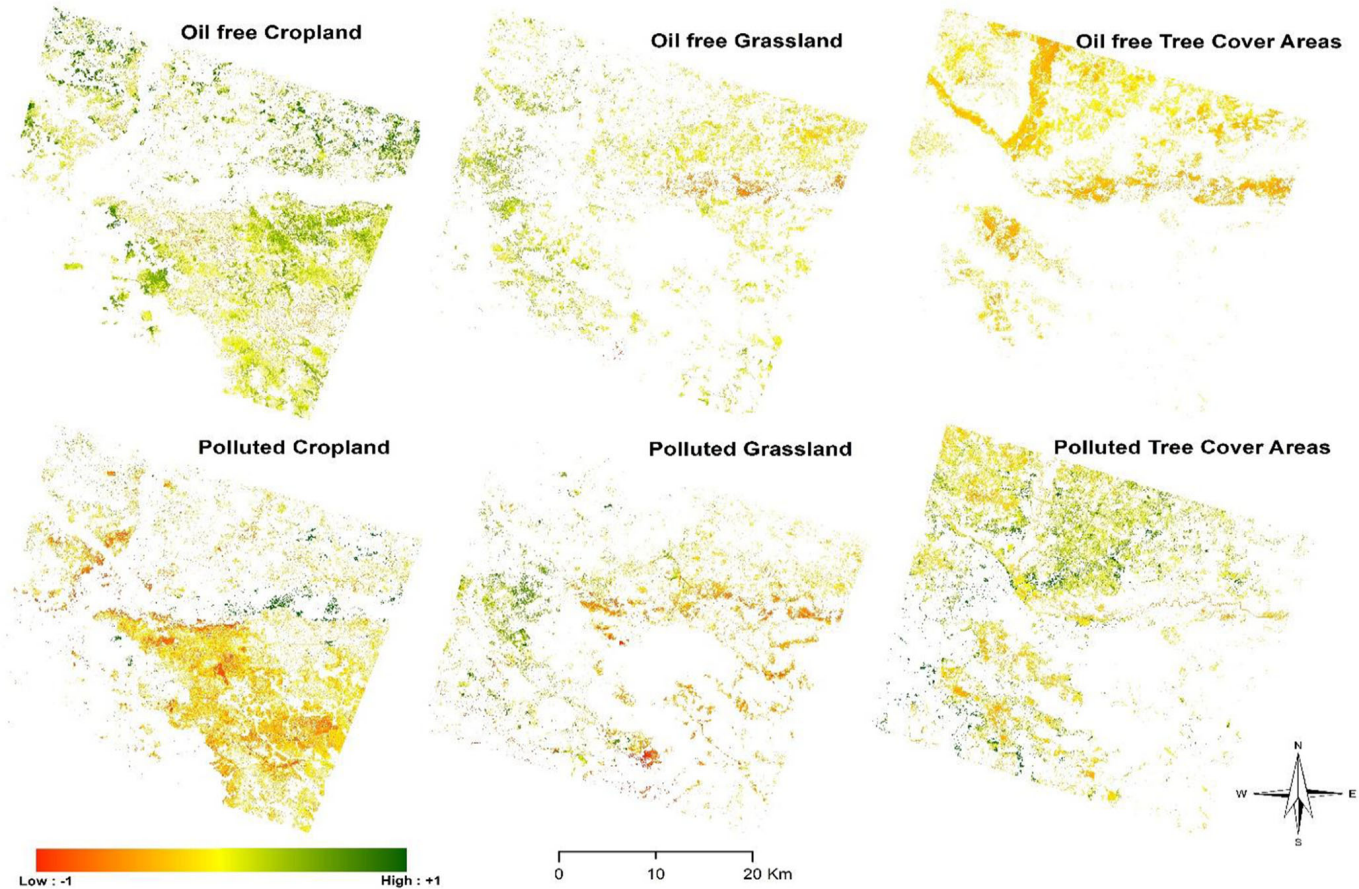
The most important variable for cropland—NDWI, grassland—SWIR, and TCA—DVI in the classification processes. Result shows that the most important variable for cropland and grassland classification had the best split into oil-impacted and oil-free vegetation, as opposed to TCA subset where the most important variable did not give favorable split into oil-impacted and oil-free TCA

In general, the moisture-related indices and sensitive bands (shortwave infrared 1) were observed to have more significant contribution in distinguishing oil polluted from oil-free landcover types both at the macro level of the entire study area and at the micro level of the individual landcover subsets. This is expected as the fundamental characteristics of stressed vegetation are their inability to carry out basic life-supporting functions such as respiration, transpiration, and photosynthesis (Arellano et al. 2015), which the classifier can rely on from the distinctions provided by the indices for class assignment. Figure 6 shows the most important variables (i.e., NDWI, SWIR, and DVI) in the classification process for cropland, grassland, and TCA landcover subsets respectively and their respective oil-free and oil polluted landcover extents.

This shows that areas with high Vegetation Health Indices and greenness are predominantly associated with oil-free landcover types especially for the oil-free cropland and grassland landcover. While areas with low vegetation health and greenness are mostly associated with polluted landcover schemes in this case the polluted cropland and grassland. However, TCA was noticed to have a poor split as indicative of the most important variable in the *RF* classification (Fig. 6). This could be associated to the fact that large parts of the Niger Delta are characterized by dense and mangrove forest vegetation (James et al. 2007), in which case the impact of crude oil would pose minimal discernible effect with a typical oil-free vegetation.

### Vegetation greenness distribution

**Fig. 7:**
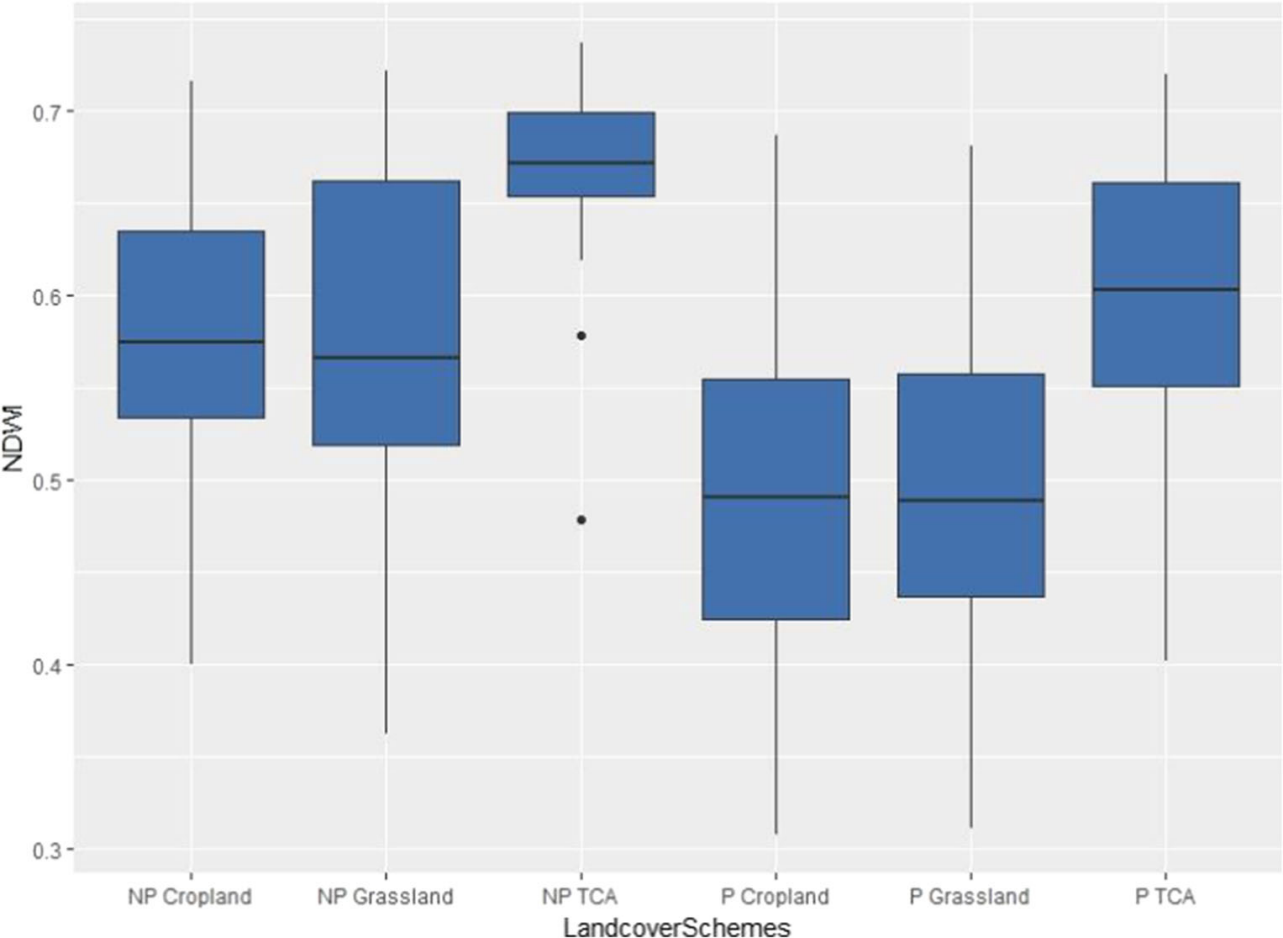
Box plot of vegetation greenness retrieved from NDWI for the polluted and oil-free landcover samples

Figure 7 is a box plot showing vegetation greenness retrieved from NDWI for the various polluted and oil-free landcover training sites. This was the most influential index when the full study area image was classified together with the Moisture Stress Index (MSI). Their performance in the classification operation further reinforces the potentials of moisture-based indices in depicting stress on vegetation. This plot showed the degree of variation in the health status of the oil-impacted and oil-free landcover classes. Non-polluted TCA were observed to have the highest NDWI compared to the non-polluted cropland and grassland. Generally, polluted grassland and cropland had the least NDWI greenness compared to their respective non-polluted classes. This is an indication that their health status could have been affected by the oil spill in those locations thereby accounting for lower health indices compared to the respective oil-free vegetation. Similarly, the distribution of the indices for the six classes shows little to no overlap between oil polluted and oil-free landcovers, a trend which could have accounted for the high performance of the NDWI in the classification process.

### Accuracy assessment

The confusion matrix generated was used to evaluate the result of the *RF* classification for the two scenarios implemented using the validation data (“Oil spill incident data”) (Table 4). The overall accuracy from the full image classification gave much lower accuracy (30.147%) compared to the result recorded from the various landcover subsets. Result from the tree cover densely forested areas gave the highest result of 70%, while the grassland and cropland subsets gave accuracies of 65% and 60.61% overall classification accuracy respectively. In terms of interclass accuracy, the result from the validation exercise showed that the highest user accuracies were obtained from the non-polluted grassland and polluted TCA with 80% from the subset classification. Similarly, the landcover classes with the highest accuracy when the full study area image was classified are the polluted and oil-free TCA classes with producer and user accuracies of 50% and 40% respectively. This is not surprising as result from the parameterization operation in Fig. 2 showed that the training sites used for classification had better characterization between polluted and oil-free dense canopies. Furthermore, the validation result obtained also showed that most of the classes that had better calibration also recorded higher accuracy. An example is in the case of TCA and grassland schemes which recorded high accuracies of above 80% out of bag error, also came out with 70% and 65% overall accuracies.

**Table 4:** Accuracy assessment result for the full study area and landcover-masked image classification

Map class	Full image classification			Masked classification		
	User’s accuracy [%]	Producer’s accuracy [%]	Overall accuracy [%]	User’s accuracy [%]	Producer’s accuracy [%]	Overall accuracy [%]
Non-polluted cropland	25	18.75	30.14	58.82	62.5	60.61
Polluted cropland	29.17	41.18		62.5	58.82	
Non-polluted grassland	18.18	20		61.54	80	65
Polluted grassland	30	30		71.43	50	
Non-polluted tree cover areas	50	40		75	60	70
Polluted tree cover areas	37.5	30		66.67	80	

### Spill-impacted vs non spill landcover spatial extent

**Fig. 8:**
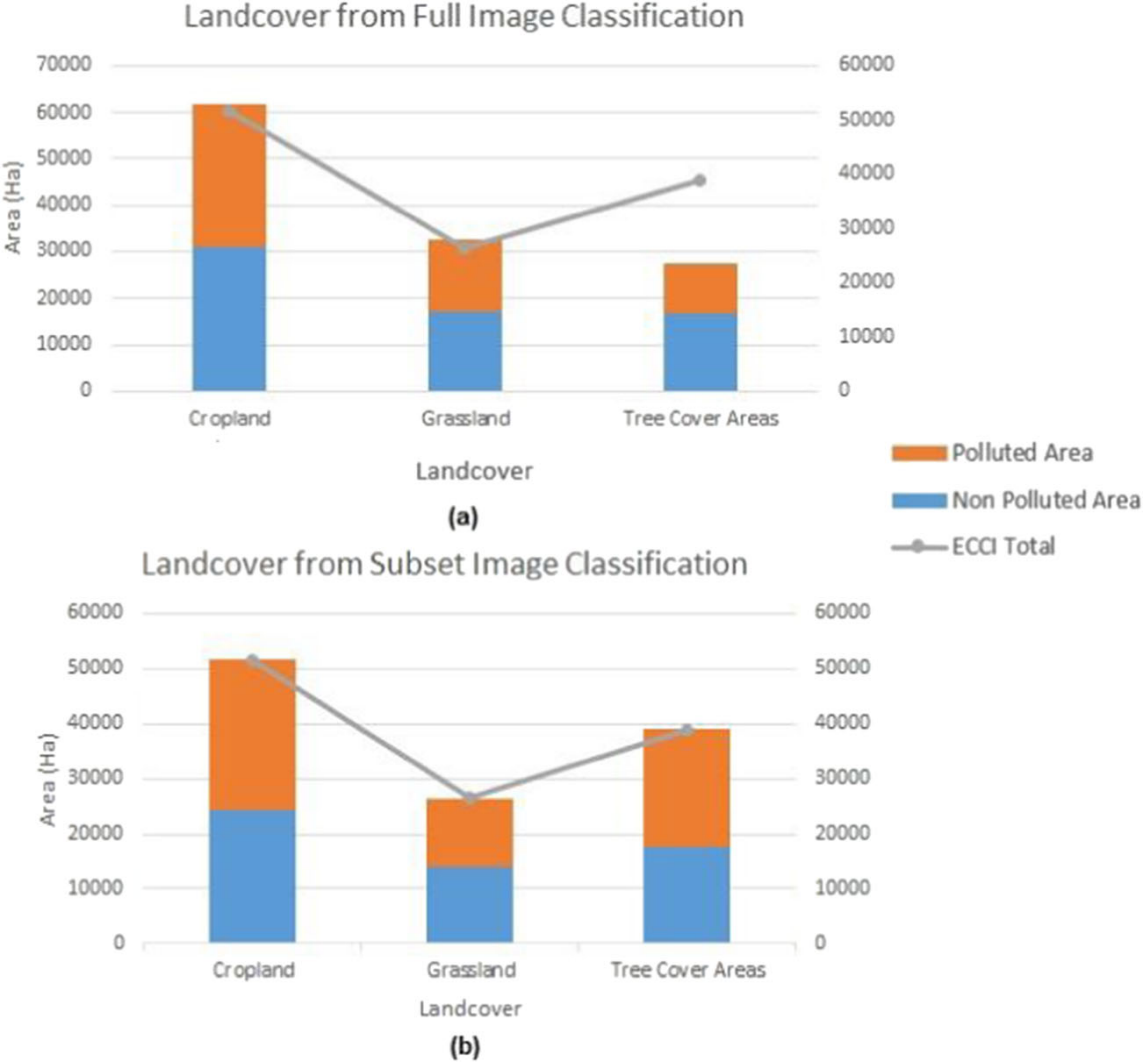
Spatial extent of oil-impacted and oil-free landcover classes retrieved from the **a** full study area image and **b** landcover subset image classification. The orange and blue stacks represent polluted and oil-free landcover classes, while the ash-colored line represents the aggregate obtained from the ECCI landcover dataset

Figure 8a and b presents a stacked bar plot comparing the total estimated area covered by oil-impacted and oil-free landcover classes from the full study area and landcover subset classification respectively. This was also compared to the total area coverage of the landcover product provided by the ECCI. Generally, the result showed that aggregated areas of polluted and oil-free landcover classes were closer to the areas from the ECCI when the image subsets are classified than when the full image is classified. Similarly, the extent of spill-impacted grassland and TCA were larger than their respective oil-free vegetation, except in cropland landcover where the area covered by oil-free cropland was larger than the oil-impacted cropland. In addition, of the six landcover classes investigated the spatial extent of oil-impacted cropland from the full study area image and cropland landcover subset image classification remained close. This, however, suggests that the spectral characteristics of the polluted cropland have remained unchanged in the two experimental classifications implemented. This is an indication that this class could have been more heavily impacted from the 2015 and 2016 spill incidents in the area.

## Discussion

Oil pollution and contamination of vegetation canopies within the Niger Delta region is a common and almost a consistent phenomenon. Few studies have focused on leveraging on the potentials of machine learning (*ML*) approaches (such as *RF*) to map the exact oil spill extent for different landcover types. This study attempted to bridge this gap by using *RF* classification to first establish the precise extent of oil spill–impacted and oil-free landcover types. Then, secondly to identify the most useful optical indicators and discriminators of oil-impacted vegetation communities from their respective oil-free vegetation. The result obtained from these experiments after calibration of sample sites and implementation of the classification operations showed that *RF* algorithm has the potential of providing reliable maps of oil-free and oil-impacted landcover. The *RF* classifier produced better results with the different landcover subsets as opposed to when the full study area image is classified, reinforcing the findings of Arroyo et al. (2010) where image space delineation for automatic classification of landcover features proved very successful.

The high calibration results obtained from the out of bag errors during the parameterization exercise of the *RF* at the micro level clearly account for the high accuracies of 70% and 65% obtained for the TCA and grassland vegetation types respectively. Although the result of the most important variable in the classification process (Fig. 6) does not mirror an excellent split as can be observed with TCA and grass landcover subsets. A major reason for this trend can be attributed to the fact that most cropland vegetations are distinctly sparse in nature and a huge volume of the oil spilt in these areas experience significant seepage into the soil sub surface and immediately causing detectable impact on crops. This invariably accounts for a better split of oil polluted and oil-free croplands, as indicative of the NDWI. Similarly, the exposed soil in cropland fields also means that much of the oiled sand surface reflective, accounting for the significant influence of shortwave infrared band (Ben-Dor et al. 1997; Cloutis 1989; Kühn et al. 2004) and its derived indices in distinguishing oil-impacted and oil-free croplands (Adamu et al. 2015; Ben-Dor et al. 1997; Brekke and Solberg 2005; Khanna et al. 2013; Kühn et al. 2004). This very much infers that biomass density could play a significant role in the characterization and mapping of oil polluted and oil-free terrestrial landcovers.

The variable importance plot obtained from the *RF* image analysis also showed that the near-infrared, shortwave infrared bands, Normalized Difference Water Index, DVI, and MSI are particularly influential in pixel class assignment. Some of these variables (shortwave infrared, MSI, and NDWI) are mostly sensitive to vegetation moisture content (Gao 1996). Several studies (Agapiou et al. 2012; Arellano et al. 2015; Benabdelouahab et al. 2015; Dotzler et al. 2015; Kalubarme and Sharma 2015) have also shown that SWIR, MSI, and NDWI variables are useful indicators of stress in vegetation canopy as a result of their sensitivity to water net loss or gain. Similarly, the NIR band is also well known for its ability to distinguish between stressed and stress-free vegetations. This is because a major characteristic of a stress-free vegetation will be the absorption of visible light for photosynthesis necessary to propagate the high reflectance of near-infrared energy (Ben-Dor et al. 1997; Knipling 1970). It is without doubt that these variables have the most ideal spectral information to characterize oil-free from oil polluted vegetation. The complex interaction of these variables is a major reason for their incorporation in the classification process basically suggesting that stress as a result of oil pollution can be better characterized and mapped.

**Fig. 9:**
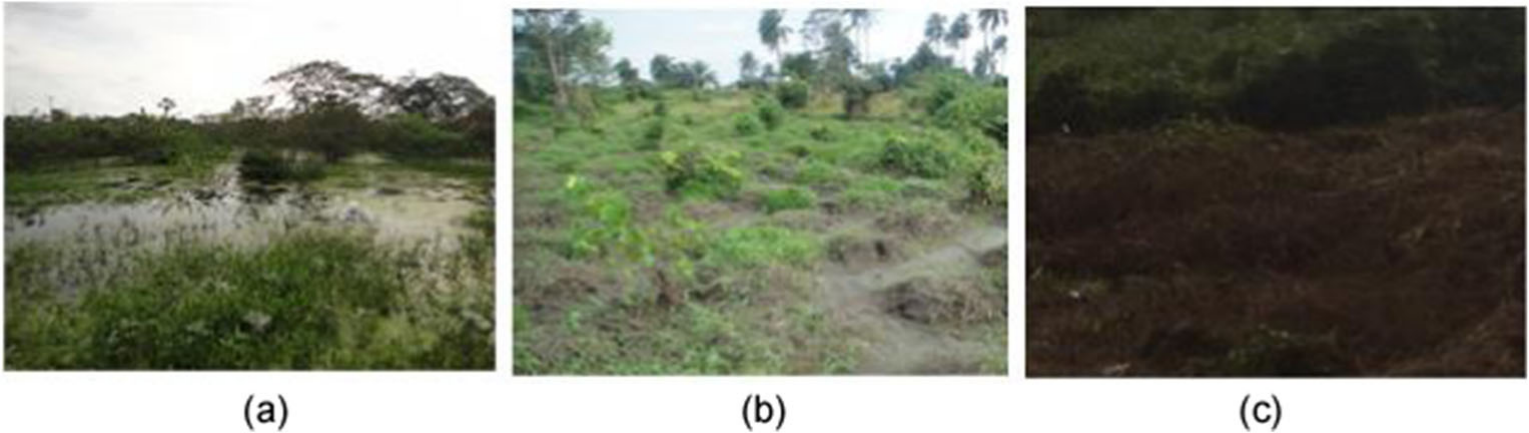
Potential influences to pixel misclassification of an oil polluted site. **a** Waterlogged areas. **b** Cleared and exposed surfaces. **c** Dried vegetated areas which often lead to burning/burn scars

In addition, the result obtained from the spatial extent of the classified maps for polluted and oil-free landcovers further suggests that cropland had the most significant impact, as the areas recorded from the full study area image and cropland landcover subset remained similar. This is quite contrary to the results obtained from the TCA and grassland landcover, where the spatial extent of their polluted landcover had a much higher area than their non-polluted/oil-free landcovers. A possible reason for this trend could be as a result of over generalization of the extent of spill-impacted landcover overlapping with other areas where vegetation stresses by other stressors exist. A post classification ground truth exercise carried out showed that features such as waterlogged areas, dried vegetation, burned vegetation, and cleared/exposed surface often exhibited similar spectral signatures as polluted sites and were classified as such. This is in line with observations made by Khanna et al. (2013) and Kokaly et al. (2013). Although most of the aforementioned misclassification anomalies are also vegetation stress related, accounting for the superior performance of the NDWI, NDVI, SWIR, and NIR in the classifications processes. Figure 9 shows some the areas that exhibited similar spectral response.

The problem of pixel misclassification in image classification is a general problem as also observed in (Ishida et al. 2018; Xiao and McPherson 2005; Zlinszky et al. 2012) where the characterization of a single vegetation type into a more narrow group by species delineation or health status has been implemented. The occurrence of pixel mismatch and over generalization of landcover spatial extent is very much apparent in this study. One way of addressing this problem in the future study is the incorporation of other relevant variables (such as radar datasets, digital elevation model, soil-type map and soil moisture) which generally do not specifically rely on the biochemical components of vegetation, rather the structural characteristics of vegetation and environmental factors are depended on to further improve discrimination accuracy.

However, the concentration and size of spill also plays a significant role in the detection and mapping of affected areas using the satellite image. Studies such as Adamu et al. (2016) have shown that the size of oil spill with respect to volume and age of oil is a major determinant of detectability of spill effect. This is largely predicated on the fact that not all spill incidents come in large sizes or quantities that can be meaningfully captured by the satellite sensors or pose detectable stress on vegetation communities. In this study, we addressed this challenge by using only spills with 1000 sqm or above in size to ensure that the characteristics of a typical spill site are reasonably captured within the spill epicenter and adjacent pixel used for classification. It was however observed that other stress factors and features with same spectral characteristics can be potentially misclassified as oil polluted landcover, which also transcend the results of the two image classification levels (micro and macro level) implemented. These certainly call for further research, especially using fuzzy techniques in establishing precise spill threshold values for adequate detection and classification purpose.

## Conclusions

This study aimed at applying *RF* in discriminating Landsat 8 image pixels of oil polluted and oil-free landcover types using published oil spill incident records as the basis for formulating training and validation sites. In addition, relevant Vegetation Health Indices and image spectral bands were fused and classified with *RF* classifier to support the discrimination process. Classification operation was implemented at the full study area (macro) level and at the individual landcover subset (micro) level. Results obtained from the latter gave a better characterization of oil-free from oil polluted landcover classes, as this produced a more generalized extent compared to the crisp and granular outputs produced from the former. Over generalization and over estimation of the oil-impacted site were observed for grassland and TCA, which can be addressed by the incorporation of other relevant variables in the classifier. In addition, the result of the variable importance showed that shortwave infrared and NDWI are significant variables in distinguishing oil polluted and oil-free landcover, especially in cropland areas. However, of the three oil polluted landcovers investigated, it is apparent that polluted cropland could have had the most significant impact due to the similar result obtained (in terms of spatial extent) from the full study area and cropland image subset classification. Similarly, the high distinctive split obtained from the NDWI (i.e., the most important *RF* variable) between the oil-free and oil-impacted cropland areas, compared to the TCA and grassland, is an indication of prolonged impact of hydrocarbon crude oil on the fragile cropland vegetation.

The result obtained from this study certainly informs on the capability of using earth observation satellite data in characterizing oil spill–impacted from oil-free areas even after several months of spill occurrence. The successful application of this method and approach to distinguishing these areas certainly reinforces the potential of assessing the intrinsic linkage between oil-induced impacts and the concomitant long-term landcover changes. This will in no doubt provide a better medium for assessing landcover change with specific recourse to oil spill incident in a typical oil spill prone area like the Niger Delta region of Nigeria. Other limitations encountered in this study such as the lack of extensive cloud-free multi-temporal optical images to establish phenological changes and implement multi-temporal based classification can be systematically addressed in future studies by incorporating radar backscatter such as the freely accessible sentinel 1 SAR images in fostering the derivation of precise area extent of the damage posed by oil pollution.
